# Acinetobacter baumannii Gastrointestinal Colonization Is Facilitated by Secretory IgA Which Is Reductively Dissociated by Bacterial Thioredoxin A

**DOI:** 10.1128/mBio.01298-18

**Published:** 2018-07-10

**Authors:** Patrick M. Ketter, Jieh-Juen Yu, M. Neal Guentzel, Holly C. May, Rishein Gupta, Mark Eppinger, Karl E. Klose, J. Seshu, James P. Chambers, Andrew P. Cap, Bernard P. Arulanandam

**Affiliations:** a Department of Biology, University of Texas at San Antonio, San Antonio, Texas, USA; b South Texas Center for Emerging Infectious Disease, University of Texas at San Antonio, San Antonio, Texas, USA; c Coagulation and Blood Research Program, U.S. Army Institute for Surgical Research, JBSA Fort Sam Houston, Texas, USA; University of Oklahoma Health Sciences Center

**Keywords:** *Acinetobacter*, bacterial virulence, gastrointestinal infection, SIgA, thioredoxin

## Abstract

Multidrug-resistant Acinetobacter baumannii is among the most common causes of infectious complications associated with combat-related trauma in military personnel serving overseas. However, little is currently known about its pathogenesis. While the gastrointestinal (GI) tract has been found to be a major reservoir for A. baumannii, as well as to potentially contribute to development of multidrug resistance, no studies have addressed the mechanisms involved in gut colonization. In this study, we address this critical gap in knowledge by first assessing the interaction between secretory IgA (SIgA), the principal humoral immune defense on mucosal surfaces, and the A. baumannii clinical isolate Ci79. Surprisingly, SIgA appeared to enhance A. baumannii GI tract colonization, in a process mediated by bacterial thioredoxin A (TrxA), as evidenced by reduction of bacterial attachment in the presence of TrxA inhibitors. Additionally, a *trxA* targeted deletion mutant (*ΔtrxA*) showed reduced bacterial burdens within the GI tract 24 h after oral challenge by *in vivo* live imaging, along with loss of thiol-reductase activity. Surprisingly, not only was GI tract colonization greatly reduced but the associated 50% lethal dose (LD_50_) of the *ΔtrxA* mutant was increased nearly 100-fold in an intraperitoneal sepsis model. These data suggest that TrxA not only mediates A. baumannii GI tract colonization but also may contribute to pathogenesis in A. baumannii sepsis following escape from the GI tract under conditions when the intestinal barrier is compromised, as occurs with cases of severe shock and trauma.

## IMPORTANCE

*Acinetobacter baumannii* is an emerging bacterial pathogen recently classified as a serious threat to U.S. and global health by both the Centers for Disease Control and Prevention and the World Health Organization. It also is one of the leading causes of combat-related infections associated with injured military personnel serving overseas. Little is known regarding mechanisms of gastrointestinal tract colonization despite this site being shown to serve as a reservoir for multidrug-resistant (MDR) *A. baumannii* isolates. Here, we establish that secretory IgA, the major immunoglobulin of mucosal surfaces, promotes *A. baumannii* GI tract colonization via bacterial thioredoxin A as evidenced through significant reduction in colonization in IgA-deficient animals. Additionally, bacterial colonization and mortality were significantly reduced in animals challenged with a thioredoxin A-deficient *A. baumannii* mutant. Combined, these data suggest that thioredoxin A is a novel virulence factor, for which antithioredoxin therapies could be developed, for this important multidrug-resistant pathogen.

## INTRODUCTION

Acinetobacter baumannii is an opportunistic pathogen that has become a significant concern for clinicians due to its high prevalence of multidrug resistance (1–5). A. baumannii isolates are intrinsically resistant to many antibiotics due to a reduced repertoire of membrane porins, naturally expressed beta-lactamases, and various efflux pumps (6). Acquired antibiotic resistance, through horizontal gene transfer, has only exacerbated the problem (1, 2, 4, 7–12). Currently, the highly toxic antibiotic colistin, which targets bacterial membranes, is often used as a treatment of last resort (5, 13, 14). Furthermore, the gastrointestinal (GI) tract colonization has been linked to development of antibiotic resistance in A. baumannii (15), presumably due to close proximity of the organism to the enormous numbers and varieties of bacteria therein, allowing direct transfer of antibiotic resistance plasmids through bacterial conjugation (16, 17). However, while the GI tract is a common site of colonization (4, 15, 18–21), there is currently a gap in our understanding of the mechanisms facilitating this colonization.

Secretory IgA (SIgA) contributes to GI tract homeostasis and protection against pathogens along mucosal surfaces (22–24). SIgA is transported across mucosal epithelia by polymeric immunoglobulin receptor (pIgR) and is comprised of a secretory component (SC) covalently bound to the Fc regions of dimeric IgA (25, 26). Both SC and SIgA interact with various antigens in a nonspecific manner due to protein glycosylation associated with both molecules (22). Additionally, SC is thought to protect the dimeric IgA molecule from both host and bacterial proteases (22); however, bacteria have developed IgA-specific proteases (27–30) as well as thiol-specific reductases produced in response to this immunoglobulin (31). Given the prevalence of A. baumannii as a mucosal pathogen, it is highly likely that it would possess similar mechanisms, although none have been described.

In this study, we set out to specifically address this critical gap in our understanding of A. baumannii pathogenesis. We found not only that SIgA contributes to A. baumannii colonization in a murine oral GI challenge model but that the organism reduces the disulfide bonds of SIgA, causing separation of SC from dimeric IgA in a process mediated by secreted bacterial thioredoxin A (TrxA).

## RESULTS

### Contribution of SIgA to A. baumannii GI tract colonization.

**FIG 1:**
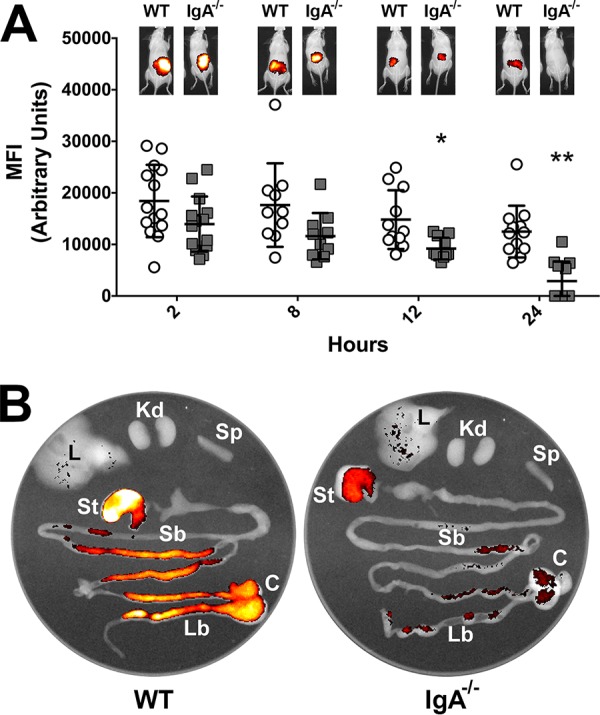
Effect of SIgA on A. baumannii clearance from the GI tract. Clearance of PSVue-794-labeled A. baumannii Ci79 was assessed in WT (white circles) and IgA^−/−^ (gray squares) mice over 24 h by *in vivo* live imaging (A). Representative images of mice following challenge are shown above the graph for each group and observation point. *Ex vivo* assessment of organs (Kd, kidney; St, stomach; L, liver; Sp, spleen; Sb, small bowel; C, cecum; Lb, large bowel) from WT (left) and IgA^−/−^ (right) mice following challenge with PSVue-794-labeled A. baumannii Ci79 was performed 24 h after challenge (B). Error bars represent ±standard deviations (SD). Statistical differences were determined by one-way ANOVA with Holm-Sidak correction for multiple comparisons (*, *P* < 0.01; **, *P* < 0.001).

Since SIgA is the primary immunoglobulin associated with mucosal surfaces, including the GI tract (22), we set out to determine if it was protective against A. baumannii GI tract colonization and infection. We first orally challenged wild-type (WT) C57BL/6 and IgA^−/−^ mice with a PSVue-794-labeled A. baumannii clinical isolate, designated strain Ci79, that we previously found to be virulent in a mouse intraperitoneal sepsis model (32). Surprisingly, *in vivo* live imaging of challenged mice revealed significantly (*P* < 0.05) enhanced A. baumannii clearance 24 h postchallenge in the absence of IgA (Fig. 1A). We subsequently humanely euthanized and collected GI tracts 24 h postchallenge from IgA^−/−^ and WT animals for *ex vivo* imaging (Fig. 1B). Again, while minimal signal was detected from GI tracts collected from IgA^−/−^ mice, fluorescence was observed throughout WT mouse GI tracts. Loss of signal in IgA^−/−^ mice could not be explained by bacterial dissemination from the GI tract, as no fluorescence was observed in surrounding tissues (i.e., liver, spleen, and kidney).

**FIG 2:**
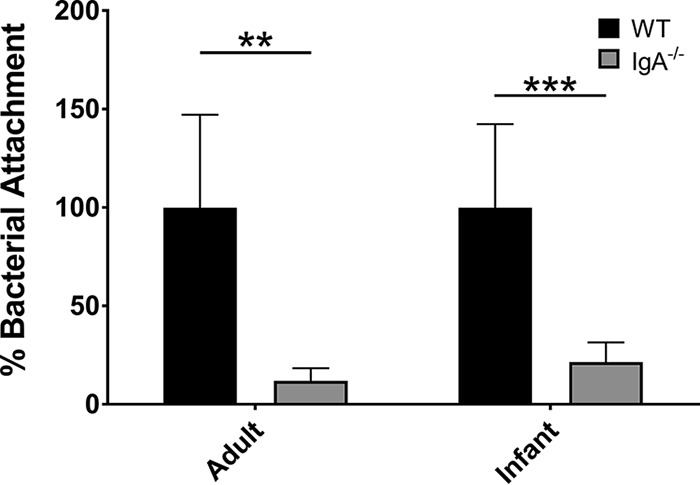
Effect of SIgA on A. baumannii attachment to the intestinal mucosa. Intestinal attachment was assessed in both adult and infant mice. The small intestine was dissected from WT and IgA^−/−^ mice. Small intestines were cut into sections measuring approximately 1 cm in length, and the intestinal lumen was exposed prior to incubation with A. baumannii Ci79 (1 × 10^8^ CFU/ml) for 30 min. After exhaustive washing to remove free bacteria, sections were homogenized and bacteria were enumerated by dilution plating. Error bars represent ±SD. Statistical differences were determined by the Welch *t* test (**, *P* < 0.001; ***, *P* < 0.0001).

We next sought to determine how IgA might contribute to, rather than protect against, GI tract colonization. Utilizing an *ex vivo* intestinal attachment assay, sections of small intestine were obtained from humanely euthanized 6- to 10-day-old mice. While lack of IgA is reported to alter intestinal microbiota (23), suckling mice lack adult microbiota, thus minimizing this confounding influence (23, 33). Additionally, although suckling WT mice do not yet produce IgA, they receive maternal IgA in milk (23). Following incubation of intestinal sections in suspensions of A. baumannii Ci79, we observed a nearly 80% reduction in bacterial attachment (*P* < 0.0001) in the absence of IgA (Fig. 2). We then repeated these experiments using sections of small intestine collected from humanely euthanized adult mice with similar results, suggesting that, in contrast to IgA, intestinal microbiota has little effect on A. baumannii attachment (Fig. 2).

**FIG 3:**
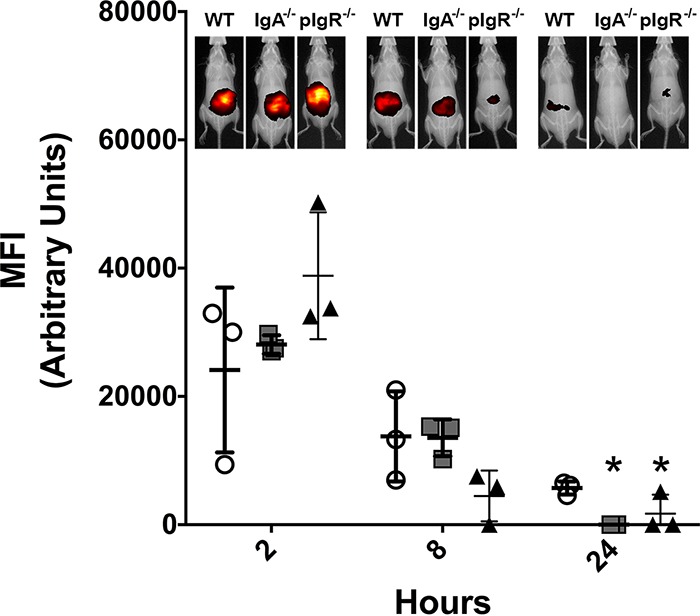
Effect of secretory component on A. baumannii gastrointestinal tract clearance. Clearance of PSVue-794-labeled A. baumannii following oral challenge was assessed in WT (white circles), IgA^−/−^ (gray squares), and pIgR^−/−^ (black triangles) mice at indicated time points over 24 h by whole-animal live imaging (MFI, mean fluorescence intensity). Error bars represent ±SD. Statistical differences were determined by one-way ANOVA with Holm-Sidak correction for multiple comparisons (*, *P* < 0.01 relative to WT). Results representative of at least two independent experiments.

While IgA^−/−^ mice lack IgA production, they still produce functional pIgR (34, 35). Furthermore, pIgR is constantly transcytosed from the basal membrane to the luminal surface of mucosal epithelial cells, where it is cleaved to produce free SC (working model; see Fig. S1, left, in the supplemental material) (25). Binding of immunoglobulin (IgA or IgM) simply speeds the process of transcytosis (Fig. S1, middle) (25). Therefore, IgA^−/−^ mice still exhibit free SC in mucous linings. Interestingly, while IgA^−/−^ mice appear to clear the fluorescent bacteria by 24 h postchallenge, clearance was even more rapid in pIgR^−/−^ mice, with fluorescent signal becoming negligible within 8 h (Fig. 3). These data suggest that free SC, rather than intact SIgA, is the primary mediator of A. baumannii GI colonization (Fig. S1, right).

10.1128/mBio.01298-18.1 FIG S1 Working model of A. baumannii GI tract colonization. The following model is based on observations from this study. A. baumannii utilizes free SC to enhance colonization of the mucous layer along the luminal surface of the intestinal epithelium (top). The host, through constant transcytosis of pIgR, generates free SC following cleavage of the receptor at the luminal surface of the intestinal epithelium (left). Binding of dimeric IgA speeds the process of transcytosis, and pIgR cleaved at the luminal surface remains associated with dimeric IgA through disulfide bonding, subsequently generating SIgA (middle). In the lumen, A. baumannii may either interact directly with free SC to facilitate mucosal colonization (low efficiency) or secrete TrxA in response to host SIgA to remove SC from dimeric IgA (top), neutralizing the protective barrier function of the immunoglobulin and providing increased concentrations of free SC to facilitate mucosal colonization (high efficiency; right). Download FIG S1, PDF file, 1.8 MB. Copyright © 2018 Ketter et al. 2018 Ketter et al. This content is distributed under the terms of the Creative Commons Attribution 4.0 International license.

### A. baumannii dissociated secretory component from SIgA.

**FIG 4:**
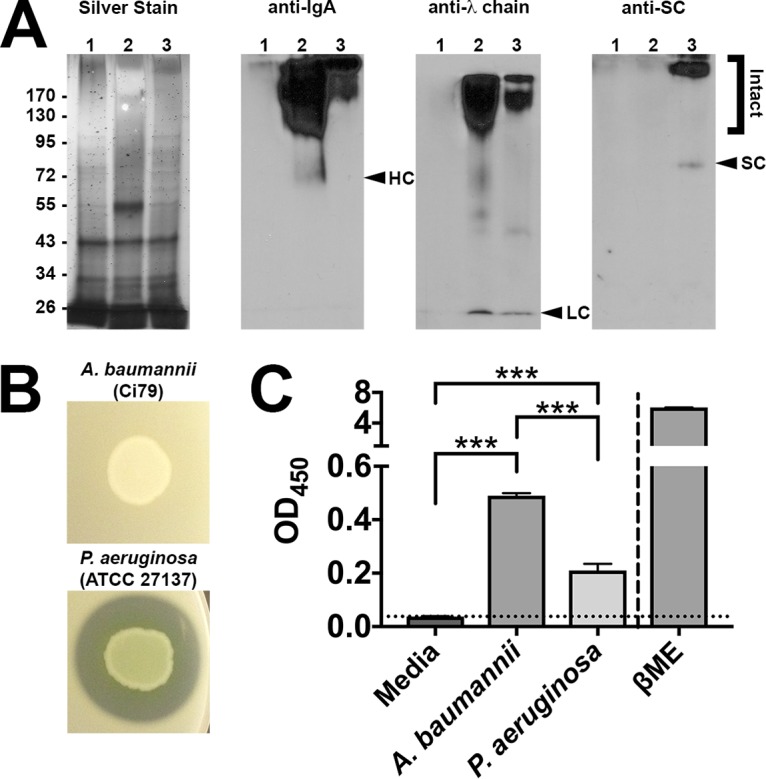
Interaction of A. baumannii with SIgA. Supernatants were obtained from A. baumannii Ci79 cultures incubated with medium alone (lane 1), serum IgA (lane 2), and SIgA (lane 3) and assessed by nonreducing SDS-PAGE (A). Gels were assessed by silver staining (far left; molecular marker sizes in kDa are shown) and Western blotting targeting the α-heavy chain (left center), λ-light chain (right center), or secretory component (far right). A. baumannii Ci79 was further assessed for secreted proteolytic activity by the development of a transparent zone on skim milk agar (B) and disulfide-reducing activity by converting DTNB to yellow TNB (C). P. aeruginosa was used as a positive protease control. β-mercaptoethanol was used as a positive reduction control. Error bars represent ±SD. Statistical differences were determined by one-way ANOVA with Holm-Sidak correction for multiple comparisons (***, *P* < 0.0001).

As many GI tract-associated bacteria produce IgA-specific proteases (27–30), we sought to assess the direct interaction between SIgA and A. baumannii by incubating Ci79 with 50 µg/ml of either human serum IgA or SIgA overnight in Dulbecco's Modified Eagle's Medium (DMEM). As expected, multiple bands were observed, indicating degradation of the immunoglobulin (Fig. 4A). However, Western blot analysis revealed clearly defined bands associated with α-heavy chain (serum IgA only), λ-light chain, and SC (SIgA only) with little smearing, indicating the absence of proteolysis (Fig. 4A). To confirm, we inoculated bacteria on LB agar supplemented with 2% skim milk to detect secreted protease (36). As expected, Ci79 exhibited no protease activity, as evidenced by lack of clearing around the bacteria (Fig. 4B). As SC is bound to dimeric IgA by disulfide bonds, release of SC from SIgA may be mediated via reductive processes. To test this possibility, we grew Ci79 (Fig. 4C), along with a variety of other A. baumannii-Acinetobacter calcoaceticus complex isolates (Fig. S2), in M9 minimal medium (M9MM) supplemented with 1 mM membrane-impermeant dithionitrobenzoic acid (DTNB) to detect secreted reductase activity (8, 37). DTNB was reduced to 2-nitro-5-thiobenzoic acid (TNB; yellow color) in the presence of the bacteria in a fashion similar to the positive reduction control β-mercaptoethanol (βME; a strong thiol-reducing agent). These data suggest that A. baumannii may break down SIgA via reductive processes targeting the disulfide bonds within the immunoglobulin.

10.1128/mBio.01298-18.2 FIG S2 Acinetobacter baumannii-Acinetobacter calcoaceticus complex isolates exhibit similar levels of attachment and DTNB reduction. Bacterial attachment was assessed for 5 unique isolates from the Acinetobacter baumannii-Acinetobacter calcoaceticus complex using excised intestinal sections collected from 6- to 10-day-old C57BL/6 mice (A). These isolates were further assessed for thiol-reducing activity utilizing the colorimetric substrate DTNB (B). Error bars represent ±SD. Download FIG S2, PDF file, 0.4 MB. Copyright © 2018 Ketter et al. 2018 Ketter et al. This content is distributed under the terms of the Creative Commons Attribution 4.0 International license.

**FIG 5:**
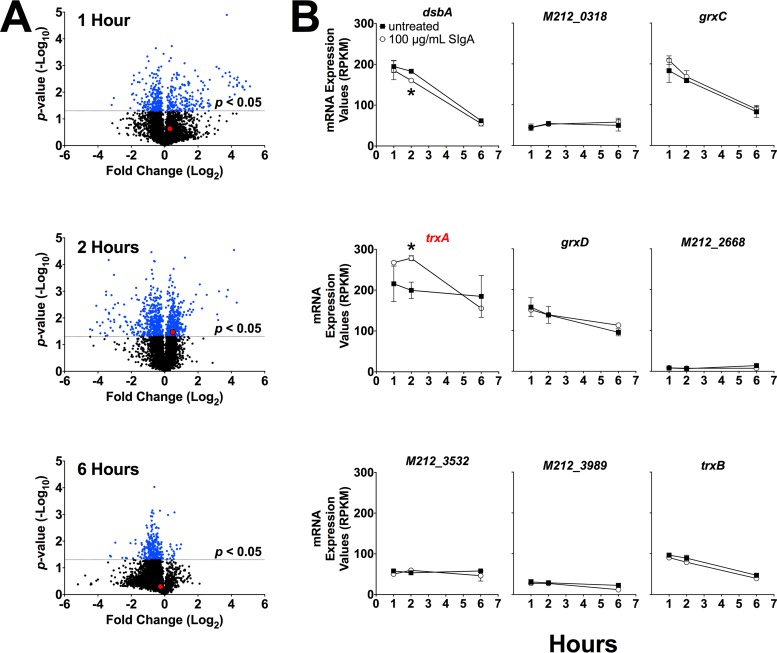
RNA-seq analysis of A. baumannii gene expression following SIgA stimulation. Global gene expression was assessed by RNA-seq in A. baumannii treated with SIgA and compared to untreated controls at 1 (top), 2 (middle), and 6 (bottom) h posttreatment (A). Gene expression of 9 thiol-reducing enzymes involved in thiol-reducing processes was directly assessed (B). Red data points and labels represent *trxA* gene expression. Error bars represent ±SD. Statistical differences were determined by Student’s *t* test (*, *P* < 0.0001).

We previously genome sequenced and annotated a series of A. baumannii isolates, including strain Ci79 (38). Utilizing BLAST2GO analysis software, we assigned gene ontology terms to annotated genes and identified those encoding proteins and enzymes involved in reductive processes (GO:005514), specifically disulfide-reducing enzymes (GO:0004791 and/or GO:0015035). These criteria identified 9 genes with characteristics necessary for reduction of disulfide bonds within SIgA (Table 1). We then incubated A. baumannii Ci79 with or without 100 µg/ml human SIgA for 1, 2, or 6 h and performed transcriptome RNA sequencing (RNA-seq) analysis. Although total gene expression was modulated at all intervals (Fig. 5A), significant modulation of disulfide-reducing proteins was observed only at 2 h. Furthermore, only *trxA* mRNA expression was significantly upregulated following SIgA exposure (Fig. 5B).

**TABLE 1:** Disulfide reductase enzymes

Gene identifier	Gene symbol	Gene name
M212_0027	*dsbA*	*dsbA* oxidoreductase
M212_0318		Dithiol-disulfide reductase^a^
M212_0534	*grxC*	Glutaredoxin
M212_0650	*trxA*	Thioredoxin
M212_2314	*grxD*	Glutaredoxin
M212_2668		Thioredoxin reductase^a^
M212_3532		Thioredoxin^a^
M212_3989		Dihydrolipoamide acetyltransferase^a^
M212_4220	*trxB*	Thioredoxin reductase

a BLAST homology only; not annotated.

### Generation of an A. baumannii thioredoxin A-null mutant.

**FIG 6:**
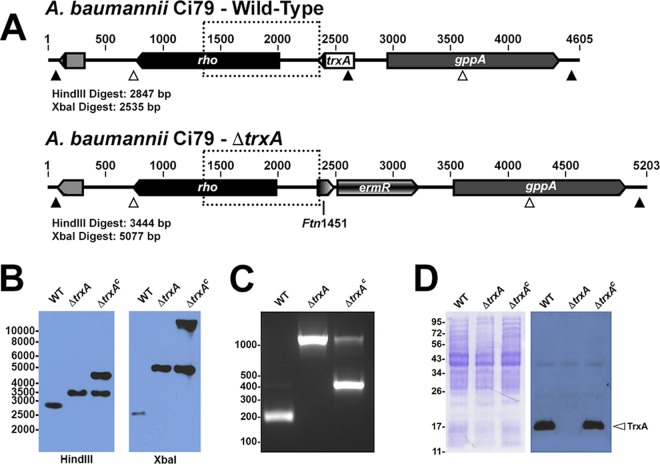
Generation of a *trxA* deletion mutant. A schematic representation of the WT A. baumannii Ci79 and Δ*trxA* mutant genome surrounding the *trxA* locus with predicted fragment sizes detected by probe following either HindIII (white arrowhead) or XbaI (black arrowhead) digestion as well as probe target region (dotted box) (A). Southern blot (B) and Western blot (C) analyses of genomic and protein extracts, respectively, from WT Ci79, *ΔtrxA*, and *ΔtrxA*^*c*^ A. baumannii were subsequently performed. PCR amplification of the *trxA* gene locus was performed using DNA from WT Ci79, *ΔtrxA*, and *ΔtrxA*^*c*^ A. baumannii isolates. The PCR amplicons were subsequently digested with SalI and subjected to agarose gel electrophoresis (C). Total bacterial proteins were separated by electrophoresis and visualized in a Coomassie blue-stained polyacrylamide gel (D, left panel) or probed with anti-TrxA antibody to detect TrxA expression (D, right panel). Molecular marker sizes for DNA (bp; B and C) and protein (kDa; D) are provided.

We next generated a *trxA* targeted gene deletion mutant (*ΔtrxA*) in WT A. baumannii Ci79. A complemented strain, *ΔtrxA*^*c*^, was also generated from *ΔtrxA*, restoring TrxA protein expression. Generation of the *ΔtrxA* strain was confirmed by Southern blot analysis showing the predicted 3.4-kb fragment resulting from HindIII restriction digest of genomic DNA, compared to the 2.8-kb fragment observed with WT DNA (Fig. 6A and B). Restriction digest of WT and *ΔtrxA* genomic DNA with XbaI also resulted in predicted DNA fragments (Fig. 6A and B), further confirming successful incorporation of the erythromycin resistance gene (*ermR*) in place of *trxA* via homologous recombination. A similar homologous recombination strategy was employed to generate the *trxA*-complemented strain (*ΔtrxA*^*c*^) with *trxA* derived from A. baumannii Ci77 containing a synonymous small nucleotide polymorphism (SNP), resulting in loss of a SalI restriction site and yet remaining 100% identical to that of the Ci79 strain on an amino acid level. While putative *trxA*-complemented strains were selected based on restoration of DTNB reduction, after several transformation attempts a single stable complement clone was obtained. However, Southern blot analysis suggested that complementation did not result in integration into the chromosome as expected, as evident by the presence of both the 3.4-kb fragment observed in the *ΔtrxA* deletion mutant and an additional band approximately 5 kb in size following HindIII digest (Fig. 6B). Similarly, XbaI digest produced both the 5.1-kb fragment associated with the *ΔtrxA* mutant and a fragment approximately 10 kb in size. Whole-genome sequencing failed to pinpoint an integration site, suggesting that the *ΔtrxA*^*c*^ strain may maintain *trxA* ectopically on a plasmid. Despite this, the presence of the Ci77 *trxA* gene in the *ΔtrxA*^*c*^ strain was confirmed through SalI digest of PCR-amplified *trxA* (Fig. 6C). PCR targeting *trxA* in the *ΔtrxA* mutant resulted in a 971-bp product corresponding to the erythromycin resistance cassette (Fig. 6C). SalI digestion of the *trxA* PCR product from WT Ci79 produced fragments of 178 bp and 199 bp in length (seen in Fig. 6C as a single band of approximately 200 bp). However, cleavage was not observed with the *ΔtrxA*^*c*^ strain, resulting in a 370-bp band. The 971-bp PCR product was observed in the complemented strain as well. Most importantly, the lack of TrxA protein expression in the *ΔtrxA* mutant and subsequent restoration in the *ΔtrxA*^*c*^ strain were confirmed by Western blot analysis (Fig. 6D).

**FIG 7:**
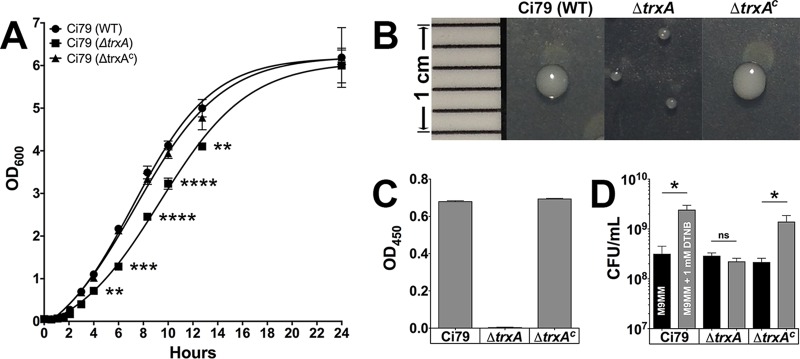
Complementation of Ci79 *ΔtrxA* with Ci77 *trxA* gene rescues phenotypic defects. The WT Ci79, *ΔtrxA*, and *ΔtrxA*^*c*^ bacteria were characterized for growth in LB broth (A), colony size on LB agar (B), DTNB-reducing activity (C), and use of DTNB for growth (D). Error bars represent ±SD. Statistical differences were determined by one-way ANOVA with Holm-Sidak correction for multiple comparisons (*, *P* < 0.01; **, *P* < 0.001; ***, *P* < 0.0001; ****, *P* < 0.00001; ns, not significant).

Deletion of *trxA* had unexpected effects on A. baumannii growth in LB medium. We observed an increase in the lag phase of growth associated with *ΔtrxA* compared to WT and *ΔtrxA*^*c*^ isolates (Fig. 7A). However, log-phase growth appeared largely unaffected. Additionally, *ΔtrxA* colonies were smaller than WT (Fig. 7B). Similar observations have been reported in yeast following disruption of thioredoxin genes and were attributed to an inability to reduce glutathiolated proteins during transition from lag- to log-phase growth (39). While the presence of secreted reductase enzymes has been reported in the extracellular proteome (40), the inability to reduce membrane-impermeant DTNB indicated that secreted redox activity was greatly reduced following deletion of *trxA* (Fig. 7C). These data suggest not only that A. baumannii secretes TrxA but that this protein is the primary thiol-reducing protein responsible for reduction of disulfide bonds in the extracellular environment. Although lacking a typical amino-terminal signal sequence, thioredoxins have been shown to be secreted by Helicobacter pylori (31) as well as various normal and tumor mammalian cells via a nonclassical pathway (41, 42).

### Effect of thioredoxin A on GI tract colonization.

**FIG 8:**
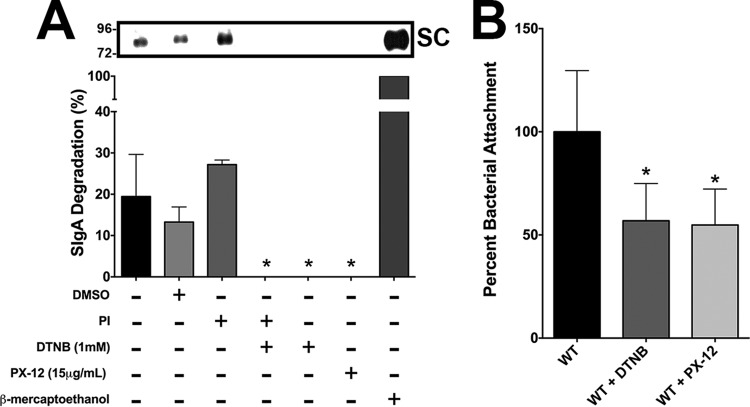
Contribution of thioredoxin to A. baumannii mucosal surface attachment. Reduction of SIgA by WT Ci79 bacteria was assessed in the presence or absence of pan-protease inhibitor (PI) and TrxA inhibitors (DTNB and PX-12) (A). Release of secretory component (SC) was assessed by Western blot analysis as a percentage of the fully reduced control (β-mercaptoethanol). Inhibition of Ci79 attachment to excised mouse intestine sections by DTNB and PX-12 was assessed by dilution plating, and bacterial attachment is expressed as a percentage compared to untreated control. Statistical differences were determined by Student’s *t* test compared to untreated control (B) (*, *P* < 0.05).

**FIG 9:**
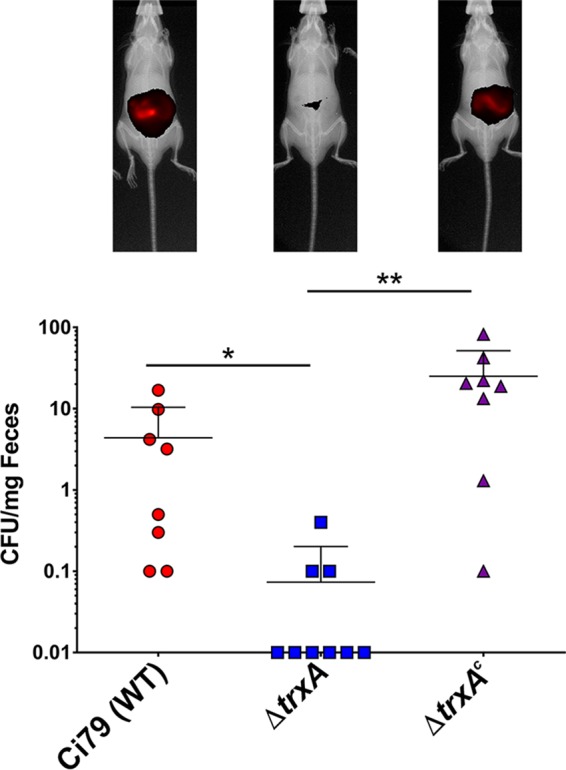
Loss of TrxA prevents A. baumannii GI tract colonization. WT C57BL/6 mice were orally challenged with 1 × 10^8^ CFU of A. baumannii Ci79, *ΔtrxA*, or *ΔtrxA*^*c*^ strain labeled with PSVue-794 for *in vivo* live imaging (images shown) or unlabeled for bacterial fecal shedding assessment. Error bars represent ±SD. Statistical differences for fecal shedding were determined by both Kruskal-Wallis test with Dunn’s correction for multiple comparisons and Fisher’s exact test (*, *P* < 0.01; **, *P* < 0.001).

An *ex vivo* intestinal bacterial attachment assay and *in vivo* oral bacterial challenge experiments were used to elucidate the role of TrxA in A. baumannii colonization in the GI tract. Two thioredoxin inhibitors, DTNB (a competitive inhibitor of disulfide reductase enzymes [43]) and 1-methylpropyl-2-mercaptoimidazolyl disulfide (PX-12; an irreversible thioredoxin inhibitor [44]), were shown to block the WT Ci79 mediated-release of SC from SIgA (Fig. 8A). Additionally, Ci79 attachment to excised intestinal sections was significantly reduced in the presence of these two TrxA inhibitors (Fig. 8B). For the *in vivo* study, mice were challenged orally with PSVue-794-labeled bacteria to monitor bacterial clearance from the GI tract via *in vivo* live imaging. A significant decrease in bacterial colonization of the GI tract was evidenced by both diminished fluorescent intensity and reduced fecal shedding of the *ΔtrxA* strain by 24 h relative to mice challenged with either the WT or *ΔtrxA*^*c*^ strain (Fig. 9). These results demonstrated the important role of TrxA in A. baumannii GI colonization. Loss of TrxA also resulted in decreased mortality following systemic A. baumannii infection. As shown in Fig. S3, while all mice succumbed to infection following a 2-LD_50_ (50% lethal dose; 1 × 10^6^ CFU) intraperitoneal challenge with both WT and *ΔtrxA*^*c*^ strains, 100% of mice challenged with equivalent doses of the *ΔtrxA* strain survived. In fact, mortality was observed only after administration of >5 × 10^7^ CFU/mouse, a nearly 100-fold increase in the associated LD_50_.

10.1128/mBio.01298-18.3 FIG S3 Loss of virulence observed following deletion of *trxA* gene expression. Groups of C57BL/6 mice were intraperitoneally challenged with various doses of A. baumannii WT Ci79, Δ*trxA*, and Δ*trxA*^*c*^ strains. Mice were monitored for mortality for 3 weeks. Download FIG S3, PDF file, 0.7 MB. Copyright © 2018 Ketter et al. 2018 Ketter et al. This content is distributed under the terms of the Creative Commons Attribution 4.0 International license.

## DISCUSSION

Data presented here suggest that SIgA contributes to A. baumannii colonization of the GI tract. We observed a significant reduction in bacterial colonization (Fig. 1 and 3) and a nearly 80% decrease in bacterial attachment in the absence of SIgA (Fig. 2). While we observed breakdown of SIgA by A. baumannii (Fig. 4A), initially suggesting possible proteolysis similar to that described with *Clostridium* spp. (27), the absence of secreted protease activity (Fig. 4B) in combination with observed secretion of thiol-reducing activity (Fig. 4C) suggested that breakdown was mediated instead by reduction of disulfide bonds within SIgA by a thiol-reducing enzyme. Indeed, prior studies have demonstrated the importance of disulfide bonds to SIgA structural stability (45). Supporting this hypothesis, Helicobacter pylori has been reported to secrete TrxA in response to host immunoglobulin for protection from the host immune system (31). Similarly, our RNA-seq analysis specifically demonstrated upregulation of *trxA* gene expression in A. baumannii following exposure to SIgA (Fig. 5). While thiol reductase activity was detected in the absence of SIgA, upregulation of *trxA* gene expression suggests that A. baumannii mounts a similar response to H. pylori. Additionally, inhibition of TrxA resulted in ablation of SIgA reduction and a significant reduction in A. baumannii attachment to the intestinal mucosal surface (Fig. 8).

Thioredoxin has been implied to have many cellular functions beyond protection from oxidative stressors, including cell cycle regulation and protein folding/transportation (46). Utilizing a tandem affinity purification (TAP)-tagged Escherichia coli TrxA, Kumar et al. (46) identified 80 TrxA-associated proteins involved in distinct cellular processes that include transcription regulation, cell division, energy transduction, and several biosynthetic pathways. Similarly, in our study, the loss of TrxA expression resulted in a multitude of effects on the bacterium, including decreased colony size and an extended lag phase (Fig. 7B). Additionally, lack of TrxA protein expression ablated secreted disulfide reductase activity (Fig. 7C). Mice challenged with A. baumannii Ci79 *ΔtrxA* also survived challenge doses nearly 100-fold higher than the LD_50_ associated with the WT Ci79 strain (see Fig. S3 in the supplemental material) and exhibited significantly increased mutant clearance and decreased fecal shedding (Fig. 9). As complementation restored all WT phenotypes, our data indicate two potential functions of TrxA secreted by A. baumannii with respect to colonization of mucosal surfaces. As shown in our working model of A. baumannii GI tract colonization (Fig. S1), TrxA secreted by A. baumannii reduces disulfide bonds within the immediate microenvironment, including those within SIgA, neutralizing protective barrier functions associated with the immunoglobulin. Second, reduced SIgA provides A. baumannii a means of colonizing mucous layers though increasing concentrations of available free SC within the mucous lining. However, the mechanism(s) of SC-mediated A. baumannii GI colonization remains elusive. While reduction of SIgA by TrxA clearly contributes to this process, immunoglobulin may not be the only target of the protein as thioredoxin also has been implicated in bacterial mucosal colonization by other mechanisms. For example, Helicobacter pylori uses secreted thioredoxin to reduce mucin molecules to their monomeric form, decreasing mucus viscosity and allowing the organism to colonize as well as facilitate migration to the epithelial surface (31). Deletion of either the TrxA or TrxC gene in H. pylori impairs the organism’s ability to colonize the stomach following oral bacterial inoculation (47).

While TrxA has now been identified as a potential mechanism by which A. baumannii colonizes the GI tract, the viability of this target for chemotherapy may be questioned due to apparent widespread distribution of TrxA gene homology among commensal bacterial species. However, the use of A. baumannii-specific TrxA as a subunit vaccine may provide at least partial protection while preventing species-nonspecific inhibition. Additionally, attenuation resulting from loss of TrxA expression by A. baumannii may allow the *ΔtrxA* mutant to serve as a useful live-attenuated vaccine strain. Current studies by our laboratory are focused on evaluating this possibility, as immunization with the *ΔtrxA* mutant provided marked protection against systemic infection with the WT strain (48).

## MATERIALS AND METHODS

### Ethics statement.

All animal experiments were performed in compliance with the Animal Welfare Act, the U.S. Public Health Service Policy on Humane Care and Use of Laboratory Animals, and the *Guide for the Care and Use of Laboratory Animals* (49). All animal work was carried out under approved protocol MU070-10/14A0 in accordance with guidelines set forth by the University of Texas at San Antonio Institutional Animal Care and Use Committee (IACUC) and the Institutional Biosafety Committee (IBC).

### Bacterial strains.

A. baumannii clinical isolate strain Ci79 was used in all experiments unless otherwise stated. Plasmids were cloned into either Escherichia coli competent JM109 cells (Promega, Madison, WI) for heat shock transformation or Top10 cells for electroporation. Other assays utilized E. coli ATCC 25922 or Pseudomonas aeruginosa ATCC 27317 (American Type Culture Collection, Manassas, VA). Bacteria were grown at 37°C to desired concentrations based on optical density of broth cultures measured at 600 nm (OD_600_).

### Mice.

Eight- to 10-week-old pathogen-free C57BL/6 mice were purchased from Charles River Laboratories (Frederick, MD). Homozygous pIgR knockout mice (pIgR^−/−^; B6.129P2-Pigr^tm1Fejo/Mmmh^) were obtained from the Mutant Mouse Resource and Research Center (University of Missouri, Columbia, MO). Male pIgR^−/−^ mice were bred with female WT C57BL/6 mice, producing litters consisting of 100% heterozygous offspring (pIgR^+/−^), ensuring that resulting offspring received SIgA in breast milk (see Fig. S4 in the supplemental material). Female pIgR^+/−^ (SIgA-producing) offspring were backcrossed with homozygous pIgR^−/−^ mice, producing litters of approximately 50% pIgR^+/−^ and 50% pIgR^−/−^ mice. Genotyping was performed by PCR utilizing the following primer combinations: pIgRKO_FW/pIgRKO_RV (Table 2) amplifying the mutant allele (150 bp) and pIgRWT_FW/pIgRWT_RV (Table 2) amplifying the WT allele (226 bp). PCR was performed with GenScript *Taq* polymerase (GenScript, Piscataway, NJ) per the manufacturer’s instructions. Homozygous IgA^−/−^ mice (34) were bred in-house for all experiments.

10.1128/mBio.01298-18.4 FIG S4 Polymeric immunoglobulin receptor knockout mouse breeding strategy. Schematic representation of breeding strategy utilized to ensure uniform exposure of infant mice to SIgA in dam breast milk. Download FIG S4, PDF file, 0.7 MB. Copyright © 2018 Ketter et al. 2018 Ketter et al. This content is distributed under the terms of the Creative Commons Attribution 4.0 International license.

**TABLE 2:** Primers used in this study

Primer name	Sequence^a^	Use
Thio-1	ACGC**GGATCC**ATGTCTGCGACTATTGTA	Recombinant protein
Thio-2	ACGC**GTCGAC**TTAAACGTTTTCGTCAAT	Recombinant protein
Up_Fw	GC**GTCGAC**CCCATATTCACCATAATCTG	SOE-PCR
Up_Rv	**GGATCC**ACTACTGGCGC**GGATCC**GGTACGGCTCCAATTTTTAG	SOE-PCR
Dn_Fw	**GGATCC**GCGCCAGTAGT**GGATCC**GACGAAAATGTTTAAG	SOE-PCR
Dn_Rv	GC**GTCGAC**TCTTCTGGGCGCTCATC	SOE-PCR
TrxA_Fw	CTAAAAATTGGAGCCGTACC	Screening
TrxA_Rv	CGTATTTTCATCTGTAACGTTACG	Screening
ABC_Fw	GTCGTAACAAGGTAGCCGTA	Strain typing
ABC_Rv	GGTGGGTTCCCCCATTCAGA	Strain typing
pIgRKO_FW	GAACTCTTGTCTTTTGTCTCC	pIgR genotyping
pIgRKO_RV	TCCAGACTGCCTTGGGAAA	pIgR genotyping
pIgRWT_FW	GAACTCTTGTCTTTTGTCTCC	pIgR genotyping
pIgRWT_RV	CTCGCCTGAATACTCCTTG	pIgR genotyping

a Boldface indicates restriction enzyme cleavage sites. Underlining indicates complementary sequence.

### *In vivo* live imaging.

Stocks of A. baumannii clinical isolate Ci79 (200-µl aliquots) were prepared from log-phase growth and frozen at −80°C in LB broth supplemented with 10% glycerol. Titers of frozen stocks were determined on three separate days and averaged. Prior to challenge, bacteria were thawed and pelleted at 3,000 × *g*. Bacterial pellets were suspended in PSVue-794 (Molecular Targeting Technologies, Inc., West Chester, PA) at a concentration of approximately 10 µl per 10^6^ CFU and placed on ice for 45 min. Bacteria were washed in TES buffer {5 mM TES [*N*-tris-(hydroxymethyl)-methyl-2-aminoethane sulfonic acid], 145 mM NaCl, pH 7.4} and brought up to a concentration of 5 × 10^8^ CFU/ml. Once prepared, mice were anesthetized by isoflurane inhalation and abdominal fur was removed using hair removal cream. Mice were challenged by oral gavage with 100 µl PSVue-794-stained bacteria and monitored at indicated intervals utilizing the Carestream MS FX Pro *in vivo* live imaging system (Bruker, Billerica, MA). At 24 h, mice were humanely euthanized and organs were excised for *ex vivo* imaging. Image analysis and annotation were performed using Carestream analysis software.

### *Ex vivo* intestinal attachment assay.

Bacterial attachment to intestinal mucosa was performed as described by Guentzel and Berry (33), with some modifications. Briefly, bacteria were washed once in sterile phosphate-buffered saline (PBS) and diluted to a concentration of 1 × 10^8^ CFU/ml. The small intestine was dissected from humanely euthanized 6- to 10-day-old infant mice, unless otherwise stated, and cut into sections measuring approximately 1 cm in length. Using fine scissors, the intestinal lumen was exposed and sections were placed in the bacterial suspension for 30 min with regular agitation. In some experiments, the bacterial suspensions were prepared with either 1 mM DTNB (Fisher Scientific, Pittsburgh, PA) or 15 µg/ml PX-12 (Tocris, Minneapolis, MN). Sections were placed in 500 volumes of sterile PBS and inverted 7 times. This was repeated once before sections were placed in 200 volumes of sterile PBS and allowed to soak for 5 min. Each section was transferred to 10 ml sterile PBS and homogenized. Dilution plating was performed on LB agar containing 50 µg/ml chloramphenicol and 10 µg/ml cycloheximide to inhibit growth of resident microbiota.

### Analysis of secreted protease activity.

Bacterial suspensions of either A. baumannii or P. aeruginosa were prepared from log-phase growth (OD_600_ of 0.7), and 10 µl was spotted onto LB agar supplemented with 2% skim milk.

### Disulfide reductase assay.

Indicated bacteria were grown at 37°C to an OD_600_ of 0.7. Each strain was pelleted at 3,000 × *g* and washed three times in M9MM (48 mM Na_2_HPO_4_, 167 mM KH_2_PO_4_, 8.5 mM NaCl, 19 mM NH_4_Cl, 2 mM MgSO_4_, 100 µM CaCl_2_, 0.4% glucose). Bacterial pellets were suspended in either sterile M9MM alone or M9MM supplemented with 1 mM DTNB and grown for 24 h at 37°C, at which point supernatants were collected. Reductase activity was observed through yellow color development detected at 450 nm.

### SIgA reduction assay.

Bacterial broth cultures were pelleted at 3,000 × *g* for 5 min, washed three times in equal volumes of sterile DMEM (Life Technologies, Inc., Grand Island, NY) to remove trace amounts of reducing substances, and suspended in an equal volume of sterile DMEM. In parallel, lyophilized SIgA from human colostrum or serum IgA from human plasma (Athens Research, Athens, GA) was diluted to a concentration of 100 µg/ml in sterile DMEM. Once prepared, 250 µl of the bacterial suspension was combined with 250 µl SIgA or serum IgA and incubated for 24 h. For experiments examining inhibition of SIgA reduction, individual preparations of SIgA were prepared containing either 2 mM DTNB or 30 µg/ml PX-12, resulting in final concentrations of 1 mM and 15 µg/ml, respectively, and incubation was reduced from 24 hours to 2 hours. Supernatants were collected and mixed 1:4 in native sample buffer (40% glycerol, 250 mM Tris-HCl, 0.015% bromphenol blue) before being subjected to nonreducing SDS-PAGE on a 12% polyacrylamide gel. Following electrophoresis, proteins were transferred to a polyvinylidene difluoride (PVDF) membrane (Bio-Rad, Hercules, CA) for Western blot analysis using goat anti-human SC primary antibody (1:1,000 dilution; Sigma-Aldrich, St. Louis, MO) followed by rabbit anti-goat horseradish peroxidase (HRP)-conjugated secondary antibody (1:3,000; KPL, Gaithersburg, MD).

### RNA-seq assay.

A. baumannii clinical isolate Ci79 was grown to log phase, and two 1-ml aliquots were obtained. Aliquots were washed three times in sterile DMEM, and the resulting pellets were suspended in either 1 ml sterile DMEM alone or 1 ml sterile DMEM with 100 µg/ml SIgA before incubation at 37°C. Bacterial cells were pelleted and snap-frozen at −80°C prior to mRNA extraction at indicated intervals using the Ambion PureLink RNA minikit (Life Technologies, Waltham, MA). rRNA depletion was performed utilizing the Ambion MICROBExpress bacterial mRNA enrichment kit (Life Technologies, Waltham, MA). Enriched bacterial mRNA was processed and subjected to RNA sequencing by Illumina HiSeq (Illumina, San Diego, CA) at the UT Health San Antonio Genomics Resource Core. Expression analysis was performed utilizing the CLCbio Genomics Workbench. Gene ontology annotation was performed on A. baumannii strain Ci79 (GI:572039789) utilizing BLAST2GO software, and genes were segregated based on functional classification.

### Generation of recombinant A. baumannii thioredoxin and anti-TrxA antibody.

A. baumannii *trxA* was amplified from genomic DNA obtained from A. baumannii Ci77 using primers Thio-1 and Thio-2 (Table 2) and subsequently cloned into pMAL-C2X, resulting in incorporation of a 3′ maltose binding protein (MBP) tag. Rosetta-gami E. coli cells were transformed with the pMAL-*trxA* construct for generation of an MBP-TrxA fusion protein (rTrxA). rTrxA expression in the transformed E. coli was induced in the presence of glucose (final concentration, 2 g/liter) and IPTG (isopropyl-β-d-thiogalactopyranoside; 1 mM) overnight in a 16°C shaking incubator. Isolation of rTrxA by amylose affinity chromatography was conducted according to the manufacturer’s recommendations (New England BioLabs, Ipswich, MA).

To generate anti-TrxA antibody, rTrxA protein was diluted to a concentration of 0.2 mg/ml and mixed 1:1 with TiterMax Gold adjuvant (Sigma-Aldrich, St. Louis, MO). BALB/c mice were vaccinated with two 50-µl subcutaneous (s.c.) injections at the base of the tail, resulting in a total dose of 10 µg rTrxA per mouse. A booster was administered 14 days after initial vaccination consisting of a single 50-µl s.c. injection of recombinant protein (5 µg). At 28 days after initial vaccination, mice were anesthetized and sera were collected.

### Generation of a targeted thioredoxin deletion mutant.

A. baumannii Ci79 DNA was extracted from a 3-ml volume of bacterial suspension using the Wizard Genomic DNA purification kit (Promega, Madison, WI). Using primer combinations Up_Fw/Up_Rv and Dn_Fw/Dn_Rv (Table 2) in combination with TaKaRa *Ex Taq* polymerase (Clontech, Mountain View, CA), a 973-bp region located directly upstream of *trxA* and a 988-bp region located directly downstream of *trxA* were amplified by PCR (Fig. S5, step 1). PCR products were processed using the Wizard SV gel cleanup kit (Promega, Madison, WI), and short overlap extension PCR (SOE-PCR) was performed under conditions described by Liu et al. (50), using primers Up_Fw and Dn_Rv and TaKaRa *Ex Taq* polymerase. The resulting PCR product (Fig. S5, step 2), containing a BamHI restriction site in place of the *trxA* gene, was subsequently ligated into pGEM-T Easy (Promega, Madison, WI) using T4 DNA ligase (Promega, Madison, WI) to make plasmid pBPA001. Transformants were identified through antibiotic selection on LB agar supplemented with 100 µg/ml ampicillin (Fig. S5, step 3). The erythromycin resistance cassette and promoter (*Fterm*^*r*^) were extracted from pKEK887 by double digest with BglII and BamHI (50). In parallel, pBAP001 was subject to single restriction digest by BamHI. A 3:1 mix of *Fterm*^*r*^ to pBPA001 was ligated to form pBPA002 and transformed into JM109 E. coli cells. Transformants were selected by antibiotic selection on LB agar supplemented with 100 µg/ml ampicillin and 100 µg/ml erythromycin (Fig. S5, step 4). A. baumannii Ci79 was grown at 37°C to an OD_600_ of 0.7 from a 1:100 dilution of an overnight broth culture. Once obtained, 5 ml was pelleted and washed in ice-cold 200 mM RbCl 3 times on ice for 5 min. Bacteria were suspended in 50 µl 200 mM ice-cold RbCl, and 20 µl (1 µg/µl) pBPA002 was added with 50 µl cryotransformation buffer (10 mM HEPES, 0.1 M CaCl_2_, 10 mM RbCl, 10% glycerol at pH 6.5) (50). The mixture was incubated on ice for 30 min and snap-frozen for 5 min in a dry-ice–ethanol bath. Bacteria were thawed at room temperature, suspended in 900 µl sterile LB, and incubated at 37°C for 90 min. Bacteria were pelleted and suspended in 100 µl sterile LB before plating on LB agar containing erythromycin. Isolated colonies were subcultured twice on selective agar and then three times on nonselective agar to ensure stable integration.

10.1128/mBio.01298-18.5 FIG S5 Plasmid construct generation. Schematic representation detailing construction of both the deletion and complementation constructs used in this study. Download FIG S5, PDF file, 0.9 MB. Copyright © 2018 Ketter et al. 2018 Ketter et al. This content is distributed under the terms of the Creative Commons Attribution 4.0 International license.

### Complementation of the deletion mutant.

The *trxA* gene was amplified from A. baumannii Ci77 by PCR utilizing the primer combination Up_Fw/Dn_Rv and TaKaRa *Ex Taq* polymerase to produce a 2,326-bp product from extracted genomic DNA (Fig. S5, step 5). The PCR product was cloned into pGEM-T to generate pBPA003 (Fig. S5, step 6). An overnight culture of A. baumannii Ci79 *ΔtrxA* was diluted 1:20 in 250 ml fresh LB and grown at 37°C to an OD_600_ of 0.8. Bacteria were placed on ice for 10 min and washed three times in sterile ice-cold 10% (vol/vol) glycerol. The pellet was suspended in 500 µl 10% glycerol, and 100-µl aliquots were frozen at −80°C. Then, 20 µl pBPA003 (1 µg/ml) was added to frozen aliquots thawed on ice. Thawed bacteria and plasmid were transferred to prechilled electroporation cuvettes (1-mm gap; Bio-Rad, Hercules, CA) and electroporated using a Bio-Rad Gene Pulser (18 kV, 200 Ω, 25 µF) (Bio-Rad, Hercules, CA). Transformed bacteria were suspended in sterile LB broth, incubated at 37°C for 1 h, and plated as before. Analysis of disulfide reductase activity revealed enhanced growth of the parental WT A. baumannii Ci79 strain in the presence of reduced DTNB, suggesting that it may utilize TNB as a carbon source (Fig. 7D). In contrast, growth of the *ΔtrxA* mutant remained static due to its inability to reduce DTNB. As such, complemented mutants were screened on M9MM agar with 1 mM DTNB. Isolated colonies were subcultured twice on M9MM plus DTNB and then three times on nonselective LB agar to ensure stable expression.

### Confirmation of genetic manipulation.

Growth was assessed through measurement of the optical density at 600 nm for A. baumannii Ci79, Δ*trxA*, and Δ*trxA*^*c*^ strains grown in LB broth lacking antibiotics over 24 h. Differences in colony size for each strain were subsequently assessed following subculture on LB agar lacking antibiotics. Genetic manipulation was confirmed by Southern blot analysis. Genomic DNAs from Ci79, Δ*trxA*, and Δ*trxA*^*c*^ strains were isolated using the GeneJET genomic DNA purification kit (Thermo Scientific, Rockford, IL), digested with XbaI or HindIII (New England BioLabs), and run on an 0.8% agarose gel at 16 V until completion. DNA was transferred to a nylon Biodyne B membrane (Thermo Scientific, Rockford, IL) and UV-cross-linked for 5 min. A DNA probe amplified from WT A. baumannii Ci79 genomic DNA using the DN_Fw/DN_Rv primer set was biotin labeled using the North2South biotin random prime labeling kit (Thermo Scientific, Rockport, IL). Hybridization and detection were performed using the North2South chemiluminescent hybridization and detection kit (Thermo Scientific, Rockport, IL) per manufacturer’s instructions. Additionally, the *trxA* gene was amplified from genomic extracts obtained from A. baumannii Ci79, *ΔtrxA*, and *ΔtrxA*^*c*^ strains with the TrxA-Fw/TrxA-Rv primer set (Table 2) and digested with SalI (Fig. 6D) to distinguish *trxA* gene origin (i.e., Ci77 or Ci79). Loss and subsequent restoration of TrxA protein expression were assessed by Western blot analysis using the mouse anti-rTrxA antibody (1:1,000) described above.

### Fecal shedding assay.

Fecal pellets were collected at 24-h intervals from WT C57BL/6 mice challenged by oral gavage with the A. baumannii Ci79, *ΔtrxA*, or *ΔtrxA*^*c*^ strain at a concentration of 1 × 10^8^ CFU per mouse. Pellets were homogenized in sterile PBS to a concentration of 0.1 mg/ml, and 100 µl was spread onto CHROMagar Acinetobacter medium (DRG International, Inc., Springfield, NJ). Fecal pellets were collected prior to challenge to ensure that the mice were not precolonized with A. baumannii.

### Statistics.

For most experiments, statistical differences were assessed by one-way analysis of variance (ANOVA) with Holm-Sidak correction or the Welch *t* test. Differences in gene expression observed by RNA-seq were assessed by Student’s *t* test. For fecal shedding, statistical differences were assessed by both Kruskal-Wallis test with Dunn’s correction and Fisher’s exact test. All statistics and graphs were compiled using GraphPad Prism statistical software.
