# Supplementary material for: Acinetobacter baumannii Gastrointestinal Colonization Is Facilitated by Secretory IgA Which Is Reductively Dissociated by Bacterial Thioredoxin A
Source: mBio. 2018 Jul 10;9(4):e01298-18. doi: 10.1128/mBio.01298-18 (PMC6050963; doi:10.1128/mBio.01298-18)
Supplement: FIG S1 [file mbo004183978sf1.pdf]

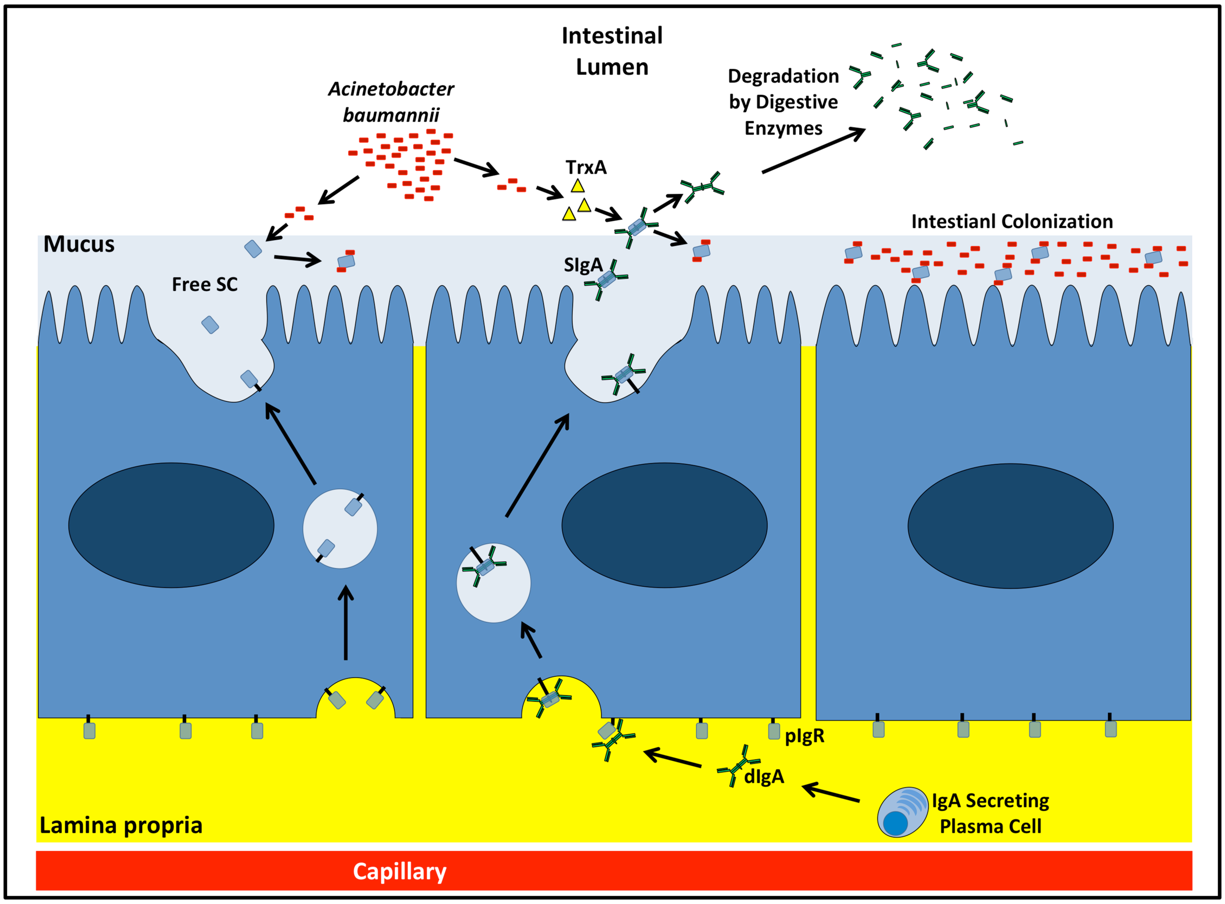


**Supplemental Figure S1: Working Model of *A. baumannii* GI Tract Colonization.** The following model is based on observations from this study. *A. baumannii* utilizes free SC to enhance colonization of the mucous layer along the luminal surface of the intestinal epithelium (top). The host, through constant trancytosis of pIgR, generates free SC following cleavage of the receptor at the luminal surface of the intestinal epithelium (left). Binding of dimeric IgA speeds the process of trancytosis and pIgR cleaved at the luminal surface remains associated with dimeric IgA through disulfide bonding, subsequently generating SIgA (middle). In the lumen, *A. baumannii* may either interact directly with free SC to facilitate mucosal colonization (low efficiency), or secrete TrxA in response to host SIgA to remove SC from dimeric IgA (top) neutralizing the protective barrier function of the immunoglobulin and providing increased concentrations of free SC to facilitate mucosal colonization (high efficiency; right).
