# Supplementary material for: Acinetobacter baumannii Gastrointestinal Colonization Is Facilitated by Secretory IgA Which Is Reductively Dissociated by Bacterial Thioredoxin A
Source: mBio. 2018 Jul 10;9(4):e01298-18. doi: 10.1128/mBio.01298-18 (PMC6050963; doi:10.1128/mBio.01298-18)
Supplement: FIG S2 [file mbo004183978sf2.pdf]

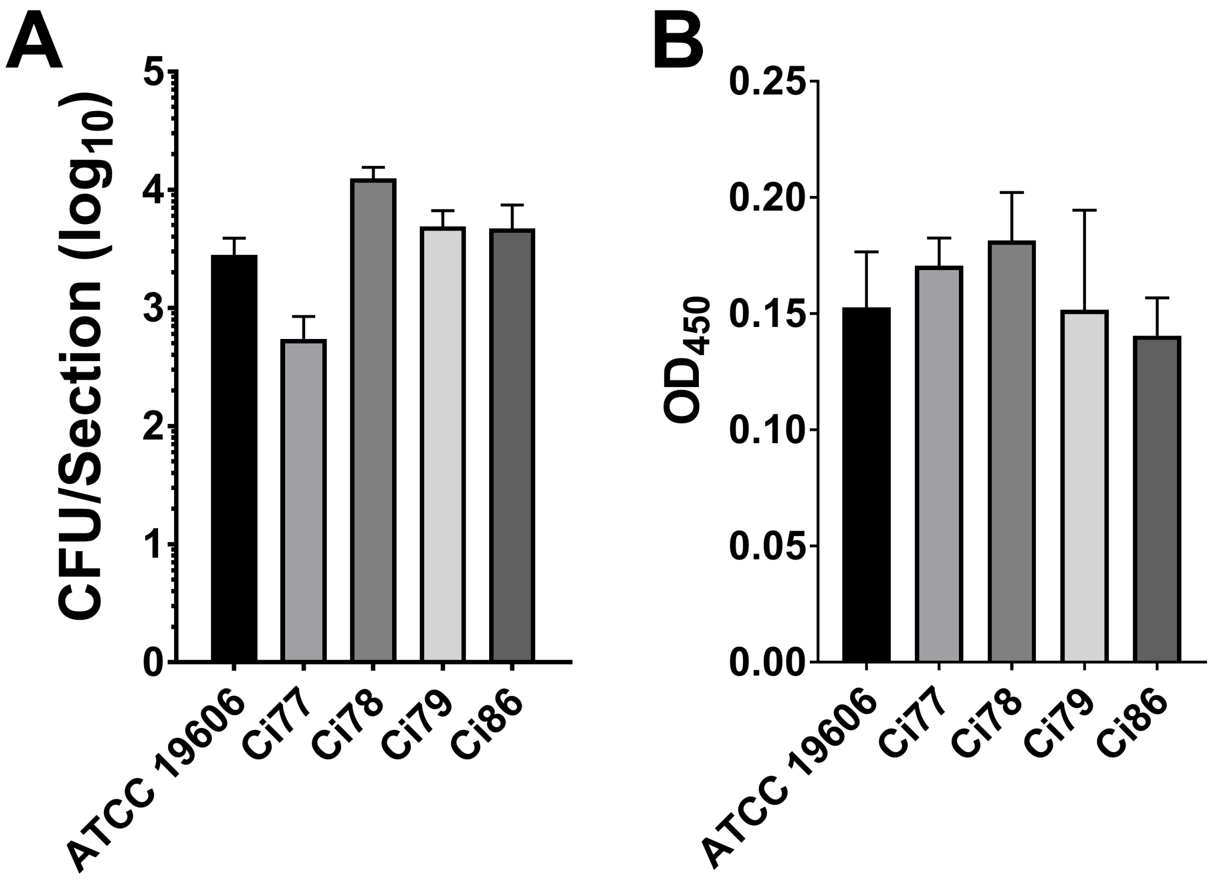

Supplemental Figure S2: *Acinetobacter baumannii - Acinetobacter calcoaceticus* complex isolates exhibit similar levels of attachment and DTNB reduction. Bacterial attachment was assessed for 5 unique isolates from the *Acinetobacter baumannii - Acinetobacter calcoaceticus* complex using excised intestinal sections collected from 6-10 day old C57BL/6 mice (A). These isolates were further assessed for thiol-reducing activity utilizing the colorimetric substrate DTNB (B). Error Bars represent ± SD.
