# Supplementary material for: Acinetobacter baumannii Gastrointestinal Colonization Is Facilitated by Secretory IgA Which Is Reductively Dissociated by Bacterial Thioredoxin A
Source: mBio. 2018 Jul 10;9(4):e01298-18. doi: 10.1128/mBio.01298-18 (PMC6050963; doi:10.1128/mBio.01298-18)
Supplement: FIG S3 [file mbo004183978sf3.pdf]

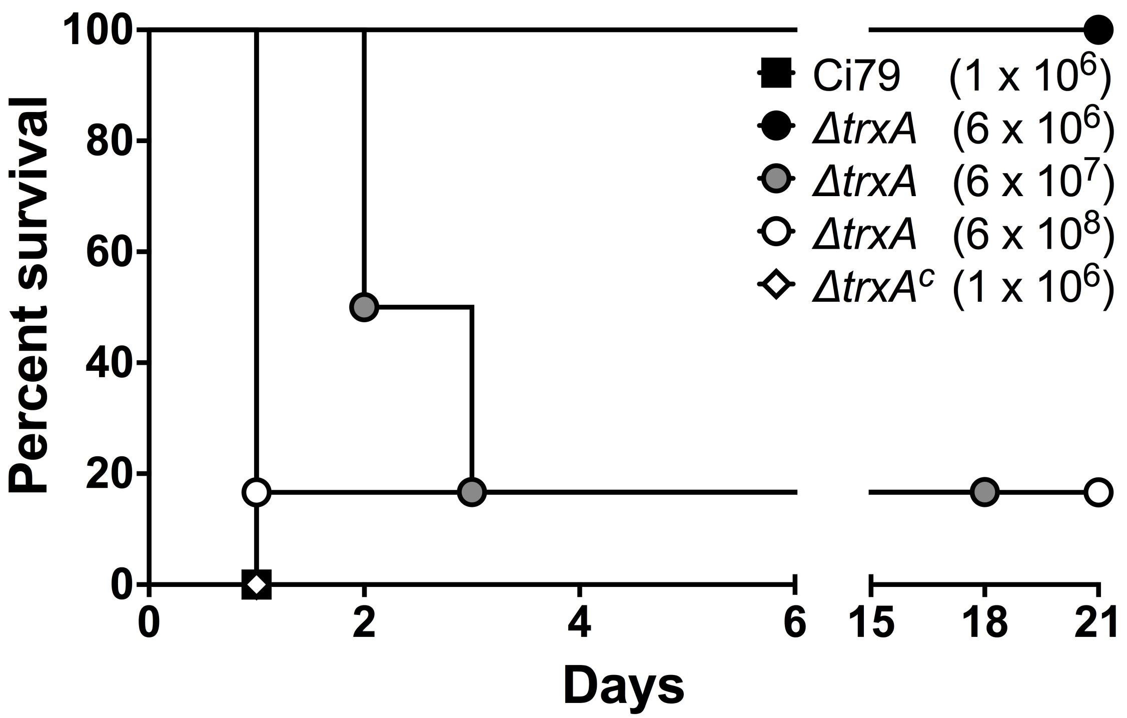

Supplemental Figure S3: Loss of virulence observed following deletion of *trxA* gene expression. Groups of C57BL/6 mice were intraperitoneally challenged with varying doses of WT *A. baumannii* Ci79, Δ*trxA*, and Δ*trxA*^{c}. Mice were monitored for mortality for 3 weeks.
