# Supplementary material for: Acinetobacter baumannii Gastrointestinal Colonization Is Facilitated by Secretory IgA Which Is Reductively Dissociated by Bacterial Thioredoxin A
Source: mBio. 2018 Jul 10;9(4):e01298-18. doi: 10.1128/mBio.01298-18 (PMC6050963; doi:10.1128/mBio.01298-18)
Supplement: FIG S4 [file mbo004183978sf4.pdf]

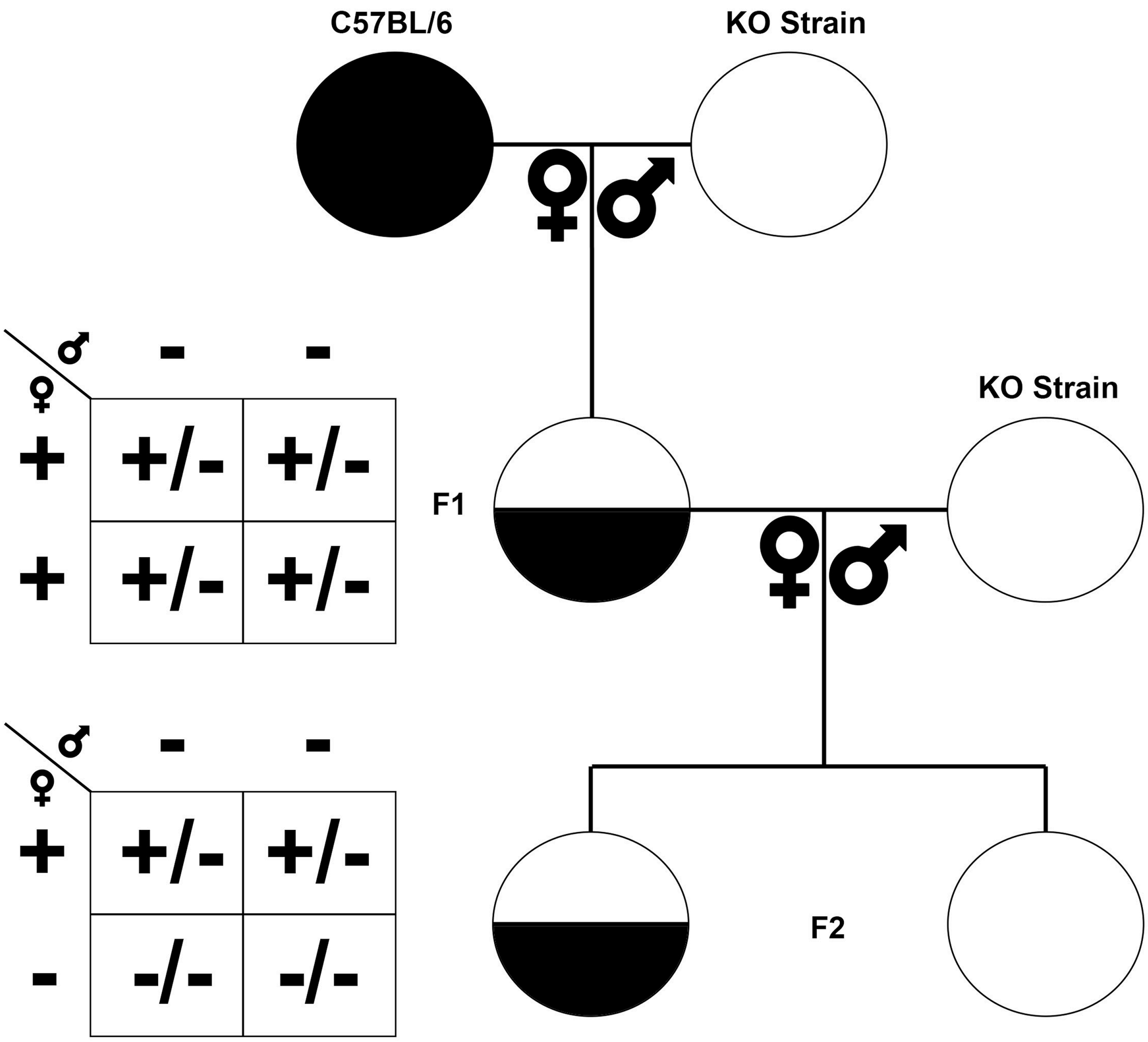


**Supplemental Figure S4: Polymeric immunoglobulin receptor knockout mouse breeding strategy**. Schematic representation of breeding strategy utilized to ensure uniform exposure of infant mice to SIgA in dam breast milk.
