# Supplementary material for: Acinetobacter baumannii Gastrointestinal Colonization Is Facilitated by Secretory IgA Which Is Reductively Dissociated by Bacterial Thioredoxin A
Source: mBio. 2018 Jul 10;9(4):e01298-18. doi: 10.1128/mBio.01298-18 (PMC6050963; doi:10.1128/mBio.01298-18)
Supplement: FIG S5 [file mbo004183978sf5.pdf]

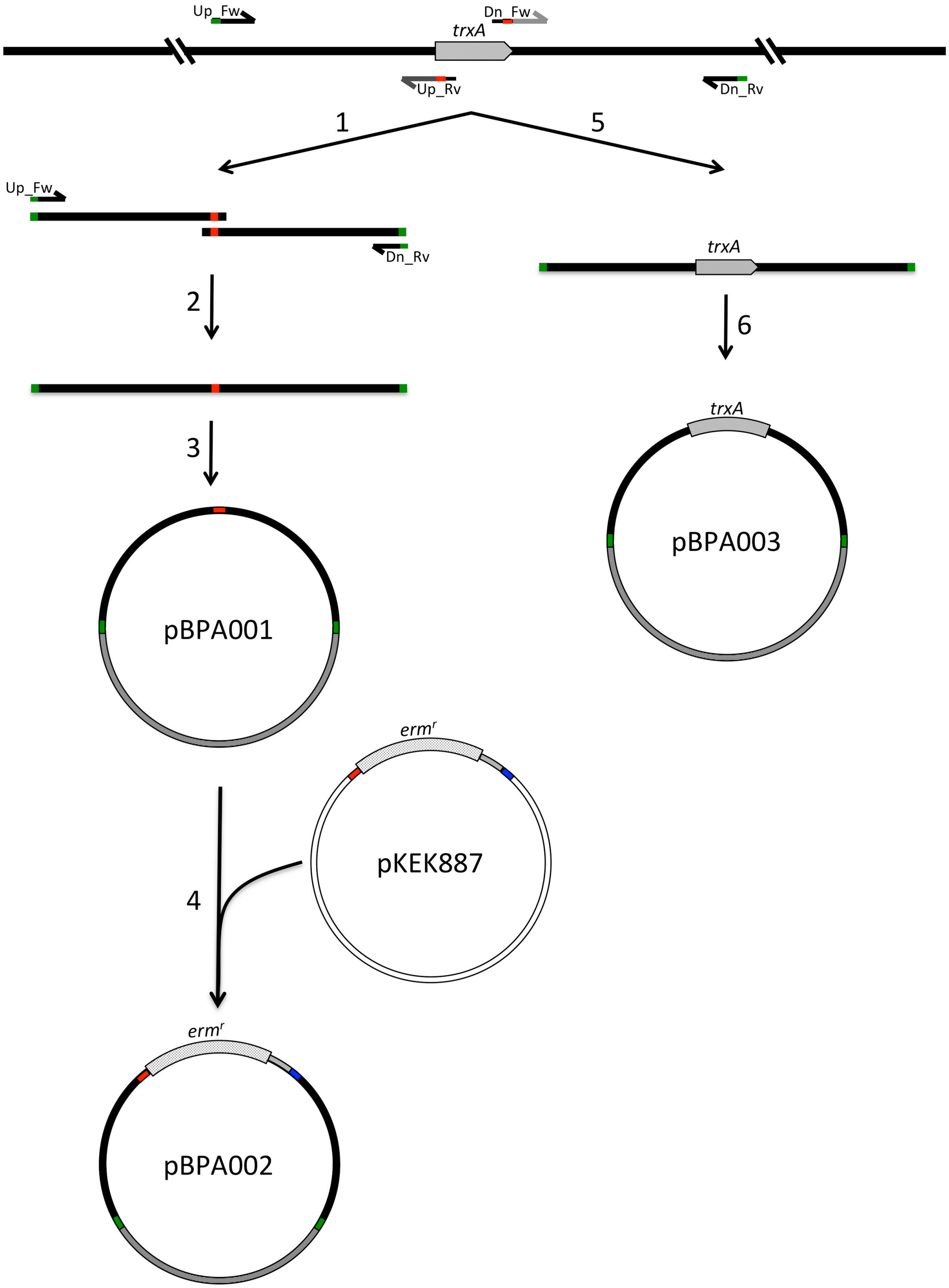

Supplemental Figure S5: Plasmid construct generation. Schematic representation detailing construction of both the deletion and complementation constructs used in this study.
